# Optimizing engagement with Internet-based health behaviour change interventions: Comparison of self-assessment with and without tailored feedback using a mixed methods approach

**DOI:** 10.1111/bjhp.12083

**Published:** 2013-12-06

**Authors:** Leanne Morrison, Rona Moss-Morris, Susan Michie, Lucy Yardley

**Affiliations:** 1 Academic Unit of Psychology, Faculty of Human and Social Sciences, University of SouthamptonUK; 2 Health Psychology Section, Institute of Psychiatry, King’s College LondonUK; 3 Clinical, Educational and Health Psychology, Division of Psychology and Language Sciences, Faculty of Brain Sciences, University College LondonUK

**Keywords:** Internet, tailoring, intervention, mixed methods, self-assessment, engagement, health

## Abstract

**Objectives:**

Internet-based health behaviour interventions have variable effects on health-related outcomes. Effectiveness may be improved by optimizing the design of interventions. This study examined the specific effect on engagement of providing two different design features – tailoring and self-assessment.

**Design:**

Three versions of an Internet-delivered intervention to support the self-care of mild bowel problems were developed that provided (1) self-assessment without tailored feedback, (2) self-assessment with tailored feedback, and (3) generic information only.

**Methods:**

A qualitative study explored participants’ engagement with each version of the intervention (*N* = 24). A larger quantitative study systematically compared participants’ use of the intervention and self-reported engagement using a partial factorial design (*n* = 178).

**Results:**

Findings from the qualitative study suggested that self-assessment without tailored feedback appeared to be less acceptable to participants because it was viewed as offering no personal benefit in the absence of personalized advice. In the quantitative study, self-assessment without tailored feedback was associated with greater dropout than when provided in conjunction with tailored feedback. There were significant group differences in participants’ engagement with the intervention and perceptions of the intervention. Self-assessment without tailored feedback was rated as marginally less engaging and was associated with fewer positive perceptions than the generic information condition.

**Conclusions:**

The acceptability of self-assessment or monitoring components may be optimized by also providing tailored feedback. Without tailored feedback, these components do not appear to be any more engaging than generic information provision.

## Background

Digital interventions have been found to be effective for a range of different health outcomes (e.g., Murray, Burns, See Tai, Lai, & Nazareth, 2005; Portnoy, Scott-Sheldon, Johnson, & Carey, 2008; Wantland, Portillo, Holzemer, Slaughter, & McGhee, 2004; Webb, Joseph, Yardley, & Michie, 2010). However, reported effect sizes differ substantially and are typically small, and interventions appear to be more effective in changing antecedents of behaviour (e.g., knowledge, self-efficacy) than actual behaviour (Bennett & Glasgow, 2009).

To understand how interventions can be optimized to improve associated outcomes, we first need to identify the mechanisms through which they may be working. A range of different design features have been used to deliver Internet-based health behaviour change interventions (see Morrison, Yardley, Powell, & Michie, 2012 for a review). Despite this, little systematic research has been undertaken to identify the specific effects on outcomes of these different ways of delivering intervention content. This means we lack understanding of which features, or combinations of features, can optimize the effect of an intervention on user engagement and health-related outcomes (Collins, Murphy, Nair, & Strecher, 2005; Collins *et al*., 2011). Employing more systematic and phased approaches to the development and evaluation of interventions may help us to identify the active design features (Bennett & Glasgow, 2009; Kraft & Yardley, 2009).

Tailoring and features to support users’ self-management of health (e.g., self-assessment, monitoring, planning, goal setting) appear to be common components of Internet-delivered interventions (Morrison *et al*., 2012). Many studies have examined the effect of tailoring, and there is growing evidence to show that online and off-line tailored interventions can be more engaging than generic interventions (e.g., Kreuter, Oswald, Bull, & Clark, 2000; Spittaels, De Bourdeaudhuij, Brug, & Vandelanotte, 2007; Strecher *et al*., 2008). For example, tailored interventions were found to evoke more positive affective responses and were associated with greater recall and use than non-tailored equivalents. To our knowledge, there have been no systematic investigations that specifically compare engagement with online interventions containing self-management features to those that do not.

Meta-analyses of online and off-line behavioural interventions provide some support for the inclusion of self-monitoring features, particularly when provided in conjunction with other features such as goal setting and performance feedback (Dombrowski *et al*., 2012; Michie, Abraham, Whittington, McAteer, & Gupta, 2009). Meta-analyses cannot, however, examine the effect of intervention features in isolation because most interventions typically include multiple features. Therefore, it is not known whether self-monitoring as an individual feature is effective or not, or whether its effect depends on the provision of other features such as tailored feedback and goal setting.

Self-assessment (i.e., the assessment of one’s own current behaviour) is a variant of self-monitoring that can be used in brief or single-session interventions when longitudinal monitoring of behaviour across multiple sessions is not feasible. Client or patient self-assessment is also incorporated as part of guided discovery and Socratic questioning in cognitive behavioural therapy (CBT) - based approaches (Wright, 2006). Given the lack of systematic research examining engagement with self-monitoring/assessment features, this study employed mixed methods to examine the effect of self-assessment when used as a precursor to goal setting. Qualitative studies can provide rich insights into what users think and feel in response to the design of interventions. However, they cannot tell us how intervention design is associated with differences in satisfaction and engagement or changes in beliefs, symptoms, and behaviour. Systematic quantitative approaches can supplement insights provided by qualitative studies (Yardley & Bishop, 2007). Mantling and factorial designs provide a bottom-up strategy for identifying the ‘active’ features of multicomponent interventions and their specific effects on outcome prior to randomized controlled trials (Danahar & Seeley, 2009; Glasgow, Davidson, Dobkin, Ockene, & Spring, 2006; Nair *et al*., 2008). These contrast with dismantling, or top-down, strategies that seek to examine the effect of particular features after a multicomponent intervention has been found to be effective.

Three different versions of ‘Gut Instincts’, an Internet-based intervention to facilitate the self-care of mild bowel problems, were developed, to allow comparison of self-assessment with tailored feedback, self-assessment without tailored feedback, and generic information. Study 1 qualitatively explored patterns in users’ engagement with the content, design, and level of interactivity provided by the intervention and facilitated interpretation of subsequent quantitative data (i.e., triangulation). A larger quantitative study compared use of and engagement with the three different versions of the intervention. Based on indications from prior meta-analyses (Dombrowski *et al*., 2012; Michie *et al*., 2009), it was hypothesized that engagement with self-assessment components will be better when tailored feedback is also provided. ‘Engagement’ refers to participants’ affective responses, the aesthetic appeal of the intervention, and participants’ perceptions of the interactivity and feedback provided by the intervention (see O’Brien & Toms, 2008 for a conceptual review).

### The intervention

‘Gut Instincts’ was developed using the LifeGuide software (Yang *et al*., 2009). It was designed to support students aged 18–25 who are experiencing mild or non-clinical bowel complaints (e.g., diarrhoea, constipation) due to their lifestyle (e.g., poorer dietary routine, increased stress, poorer sleep routines) and who may have limited experience of independently self-caring for health problems. The content was adapted from a CBT-based intervention for patients diagnosed with irritable bowel syndrome (Everitt *et al*., 2010; Moss-Morris, McAlpine, Didsbury, & Spence, 2010).

‘Gut Instincts’ was delivered in two sequential parts. Part 1 provided advice on symptoms, the digestive system, diet, diarrhoea, and constipation. Part 2 provided advice on physical activity, sleep, stress, and thoughts. Participants were invited to ‘leave a week or at least a few days’ between completing Part 1 and Part 2 to try out the practical advice suggested. However, to minimize attrition, participants who were keen to complete the intervention in one session were given the option to continue directly to Part 2. A ‘tunnelled’ architecture (Danaher, McKay, & Seeley, 2005) was used to ensure that all users were introduced to each topic in the same order – participants could not revisit particular topics of the intervention.

Three versions of ‘Gut Instincts’ were developed that were based on identical content. Two design features were systematically varied between each version – tailoring and self-assessment (see Table 2009 for a detailed summary).

**Table 1:** Summary of design differences between each condition of ‘Gut Instincts’

Self-assessment without tailored feedback	Self-assessment with tailored feedback	Generic information
Feature 1: Self-assessment		
Quizzes introduced as a tool to help participants *think* about how their thoughts or behaviours may be affecting their bowel problems (e.g., ‘taking this quiz will help you to think about…’)	Quizzes introduced as a tool that would *identify* for participants whether their particular thoughts or behaviours may be contributing to their experience of bowel problems (e.g., ‘taking this quiz will help you to find out…’)	Contained no self-assessment quizzes Contained no goal setting options
Participants required to select goals based on the information and advice provided	Participants required to select goals based on the information and advice provided	
Feature 2: Tailored feedback		
Completion of the quiz followed by a generic feedback page that re-iterated the purpose of the quiz but did not offer any personalized feedback	Completion of the quiz followed by personalized feedback page tailored to participants’ individual quiz responses	No tailored feedback provided – all participants received identical informational content
All participants saw exactly the same list of goals. These were not tailored to participants’ individual quiz responses	Participants only saw further information and advice about a topic if their individual quiz responses suggested that this would be relevant	Participants’ symptoms referred to using generic terms, for example ‘stomach or bowel problems’
Participants’ symptoms referred to using generic terms, for example ‘stomach or bowel problems’	Participants only saw the particular pieces of information and advice that were relevant to their particular symptoms, thoughts, or behaviours as indicated by their quiz responses, for example if participants reported experiencing constipation but do not include fibre within their diet, they were presented with further information on the benefits of consuming fibre. If participants reported experiencing constipation but already include fibre within their diet, they were not presented with this information	
All participants received identical informational content. Information and advice pages were not tailored to participants’ individual quiz responses	Personalized based on participants’ reported symptoms. The terms ‘diarrhoea’, ‘constipation’, or ‘diarrhoea and constipation’ were used in place of the generic term ‘stomach or bowel problems’	
	Participants only presented with goal options that were relevant to their particular symptoms, thoughts, or behaviours	

Participants in the two self-assessment conditions were asked to complete a self-assessment quiz at the beginning of each topic. Participants were then presented with a series of information and advice pages relevant to that particular topic. Participants in the tailored feedback condition only saw further information and advice about a topic if their individual quiz responses suggested this would be relevant. The informational content was identical for both the self-assessment conditions; however, the amount of information received by participants in the tailored feedback condition was adapted to their quiz responses. At the end of each topic, participants were asked to select at least one goal from a pre-defined list of suggestions (e.g., ‘to develop a mealtime routine I will eat at the same time each day’). Participants in the tailored feedback condition were only required to select goals if their quiz responses had suggested that further information on a topic was needed. Participants in the generic information condition were not asked to complete any self-assessment quizzes or select any goals. They were simply presented with a series of advice pages on each topic that were identical to those in the self-assessment without tailored feedback condition.

## STUDY 1: QUALITATIVE STUDY

## Method

### Participants and procedure

Study 1 took place between June and November 2010 and was approved by the appropriate ethical bodies. Sixteen females and eight males aged 20–54 (*Mdn* = 25 years) with experience of non-clinical or mild bowel complaints were recruited to the study using paper-based advertisements and snowballing techniques. The sample was predominantly educated to degree level (83%) and were regular Internet users (*Mdn* = 4.5 hr/day).

Interviews comprised a set of questions about participants’ experiences of bowel problems, then a think-aloud interview to discuss their thoughts about the intervention whilst they were using it, and finally further general questions about their experiences of using ‘Gut Instincts’ and the Internet (see Table 2006). Participants were explicitly encouraged to share any critical thoughts about the intervention. Participants were not informed that there were three different versions of the intervention or what version of the intervention they were using prior to the interview. For piloting purposes, participants were required to complete the self-report measures used in Study 2.

**Table 2:** Interview questions and prompts used to guide the semi-structured interviews

Participants’ experience of stomach or bowel problems
How would you describe your stomach or bowel problems?
How serious do you think your stomach or bowel problems are?
How does having stomach or bowel problems make you feel?
What do you think causes your stomach or bowel problems?
Can you tell me about how you deal with your stomach or bowel problems?
What would help you to deal with your stomach or bowel problems better?
Can you tell me about any ways your lifestyle may have changed because of your stomach or bowel problems?
Neutral prompts used during the think-aloud interview
What are you thinking now?/What are your thoughts about this page?
Can you tell me more about that?/Why is that?
How are you feeling?/How do you feel about that?
Why did you do that?/Why did you click on that?
That’s really interesting/That’s really useful, thank you
Participants’ experience of using ‘Gut Instincts’ and the Internet
How did you feel about using this website?
How would you decide whether to use this website for the first time?
How would you decide whether to use this website again, for a second or a third time?
How do you feel about the information and advice on this website?
How do you think the website could be improved?
What didn’t you like about it?
What did you like about it?
What was missing from this website?
If there were a number of websites like this one, how would you decide which one to use?
When do you think using this website would be helpful?
When do you think using this website would not be helpful?
Can you tell me about any experiences you’ve had using health-related websites?

### Data analysis

Inductive thematic analysis was used to identify recurring patterns and themes within the data (Braun & Clarke, 2006), following guidelines for establishing validity in qualitative research (Yardley, 2008) and incorporating techniques from grounded theory (e.g., open *in vivo* coding, constant comparison, deviant case analysis, memo writing, and diagramming, Charmaz, 2006; Strauss & Corbin, 1990). All transcripts were coded by the first author who developed a preliminary list of codes using participants own words wherever possible, which were then organized into a coding framework. This coding framework was discussed with and agreed upon by the fourth author. All 24 transcripts were coded using this framework making modifications when needed. The frequency with which each theme occurred is reported qualitatively (e.g., stating that a view is expressed by a ‘few’, ‘some’ or ‘most’ participants). Exact frequencies are not reported, as how often a theme is expressed cannot be taken as a reliable measure of how prevalent or important that view is in the target population.

## Results

For the purposes of brevity, only themes relevant to the design differences between the three conditions have been presented. Perceptions of goal setting will not be discussed because this did not evoke strong or varied reactions from participants in the two self-assessment conditions. Please refer to Morrison (2012) for the full analysis.

### Perceptions of the information and advice

Self-assessment quizzes appeared to create an expectation for personalized feedback. Participants in the self-assessment without tailored feedback condition expressed disappointment at receiving generic information and advice. For one participant, this meant that the self-assessment quizzes were not worth completing because careful consideration of responses did not actually change the subsequent information and advice received.

I think it would be nice to have like a combination of it telling me why um like what I am give me a name it’s just something a bit more fun like call me a restless sleeper um it just makes it more personal it just makes me feel like there was a reason for me doing the quiz otherwise I’ve just done it for nothing kind of thing.(Self-assessment without tailored feedback.)

Participants in the tailored feedback condition considered tailored advice to be authentic because it was able to provide interesting and useful insights into one’s characteristics or behaviours. However, the authenticity of tailored advice was undermined if it was perceived to be incorrect or inappropriate. A few participants felt that their tailored feedback was not consistent with their self-assessment quiz responses, either because they perceived the tailored feedback to be wrong (e.g., they received advice on changing a behaviour they believed they did not perform) or because the tailored feedback did not fit with their own understanding of their symptoms (e.g., the tailored feedback over-emphasized the impact of their bowel problems).

But it did kind of make me feel like the tailoring was a fraction off [interviewer: Ok] Because it hadn’t registered that I hadn’t said that I'd said no I don’t do that [interviewer: And how did you feel when that happened?] Um, you there is there is a kind of tendency to think well in that case is this information for me because I don’t do that …. (Self-assessment with tailored feedback.)

Many participants had difficulty selecting responses to the self-assessment quizzes because the available responses did not accurately describe their particular thoughts or behaviours. Consequently, participants in the tailored feedback condition appeared to anticipate that the advice would not then be appropriate for their particular personal circumstance as they had not provided adequate or accurate information on which to base it.

I haven’t given that much detailed information to really warrant a proper prognosis I don’t think personally … I think they’re sort of jumping to conclusions about stuff at the moment. (Self-assessment with tailored feedback.)

I kind of disagree crazy quiz what are you telling me I dunno maybe I put sometimes a bit too often but yeah it’s hard to know whether it’s sometimes or rarely. (Self-assessment with tailored feedback.)

### Perceptions of the self-assessment quizzes

Participants reported that they were happy to expend effort answering the intervention’s questions if they could see a clear personal benefit for doing so, such as receiving personalized feedback, receiving more in depth information, a welcome interaction to break up the text, or an indication that the intervention cared about their personal thoughts, feelings, and behaviours. Participants in the self-assessment without tailored feedback condition, who did not receive any personalized feedback, expressed the strongest dislike for the self-assessment quizzes.

[Interviewer: can you tell me more about why you decided to find out more?] [about thoughts and feelings] Yeah I dunno it’s getting like I feel like I’m spiralling into something really sort of exciting and interesting it’s almost like the website cares what I think no-one asks me these questions but the website does. (Self-assessment with tailored feedback.)

I don’t like answering questions as um I dunno I find it like waste of time. (Self-assessment without tailored feedback.)

Several participants also reported that being directed to think about one’s thoughts and behaviours helped them to feel more confident about managing their bowel problems. However, a couple of participants did suggest that answering quizzes can make symptoms seem more serious than they had previously considered them to be.

Yeah, ah when I look at that I suddenly feel like the problem’s far worse than I think it is [laughs] [Interviewer: and how does that make you feel?] Um I don’t know it’s one of those things I suppose I look at it psychologically that if I don’t concentrate on it, it isn’t a problem [laughs]…. (Self-assessment with tailored feedback.)

Engagement with the intervention appeared to be perceived by participants as a reciprocal interaction; the more you give the intervention (by answering questions), the more it can give back to you in return (detailed personalized information). Participants seemed happy to answer questions if they had gained earlier benefits or, at the very least, were clear at the start what the personal benefits to answering them would be. Equally, participants appeared to be more inclined to answer study measures if they had found the intervention helpful.

Because yeah I think I’ve got what I needed out of the site so now it’s just like giving something back to the site so this is more the sort of am I feeling like I should um give something back maybe reciprocate the favour you’ve helped me out website now should I help you out answering these questions. (Self-assessment with tailored feedback.)

## STUDY 2: QUANTITATIVE STUDY

## Method

### Participants and procedure

The study took place between November 2010 and May 2011 and was approved by the appropriate ethical bodies. Study advertisements were sent by email to 1,408 departments in 106 universities throughout the United Kingdom to target students who experience non-clinical or mild bowel complaints. Email invitations were followed up by telephone (1) to confirm that the invitation had been received and (2) to prompt distribution amongst students (i.e., to ensure that the initial email was not ignored). Advertisements were also placed on various social networking sites. After registering for the intervention, participants (*N* = 553) were automatically allocated by the LifeGuide software to one of the three versions of ‘Gut Instincts’ using simple randomization. Participants who completed the intervention (i.e., saw both parts of the intervention and answered the follow-up measures, *n =* 178) were entered into a prize draw to win £100 (see 2011 for sample characteristics). No formal screening of participants’ self-reported symptoms was conducted. Quiz responses from participants in the two self-assessment conditions (*n* = 311, 81%) indicated that the majority of participants (*n* = 303, 97%) reported experiencing symptoms consistent with diarrhoea and/or constipation.

### Measures

*Intervention usage* was automatically recorded using the LifeGuide software (Yang *et al*., 2009), including total duration spent looking at ‘Gut Instincts’, duration on each topic, and responses to self-assessment quizzes.

Self-reported *positive perceptions* of the intervention were measured online at follow-up (i.e., immediately after completion of Part 2 of the intervention) using the Positive Intervention Perceptions Scale (PIPS, Yardley *et al*., 2010). This measured the extent to which participants perceived that the website gave them all the advice they needed, was helpful, and was trustworthy.

Self-reported *engagement* was measured online at follow-up using the Website Evaluation Questionnaire (developed by the first and fourth authors, see Appendix^2009^). It was designed to measure participants’ evaluation of the specific design features added to the generic information. Reliability was examined in a pilot sample of 15 participants. It comprises three scales: perceptions of personal relevance (measures the extent to which participants perceived the intervention to be tailored; α = .96), perceptions of self-assessment and goal setting (measures the extent to which participants perceived that the intervention helped them to reflect on their current behaviours and set goals to ease their symptoms; α = .89), and engagement (measures the extent to which participants perceived the intervention to be attractive and enjoyable to use; α = .95).

*Demographic characteristics* were measured at follow-up. Participants’ age, sex, educational status (studying for or had a university degree), and frequency of Internet use (hr/day) were recorded. This measure was optional.

### Statistical analyses

Statistical analyses were performed using SPSS 16.0 (SPSS Inc., Version 16.0. Chicago, IL). The proportions of participants dropping out of each condition were compared using the two proportion *z*-test. Group differences in the proportion of dropout at different points in the intervention, sex, and education status were compared using chi-square analyses. Group differences in time spent using the intervention, age, frequency of Internet use, and positive intervention perceptions were compared using separate one-way independent ANOVAs. Group differences in perceptions of personal relevance, self-assessment and goal setting, and engagement were compared using one-way MANOVA to control for the intercorrelations between the summary scores on each of these scales. *Post-hoc* analyses were conducted using Tukey’s HSD test.

## Results

### Attrition and exposure

**Figure 1:**
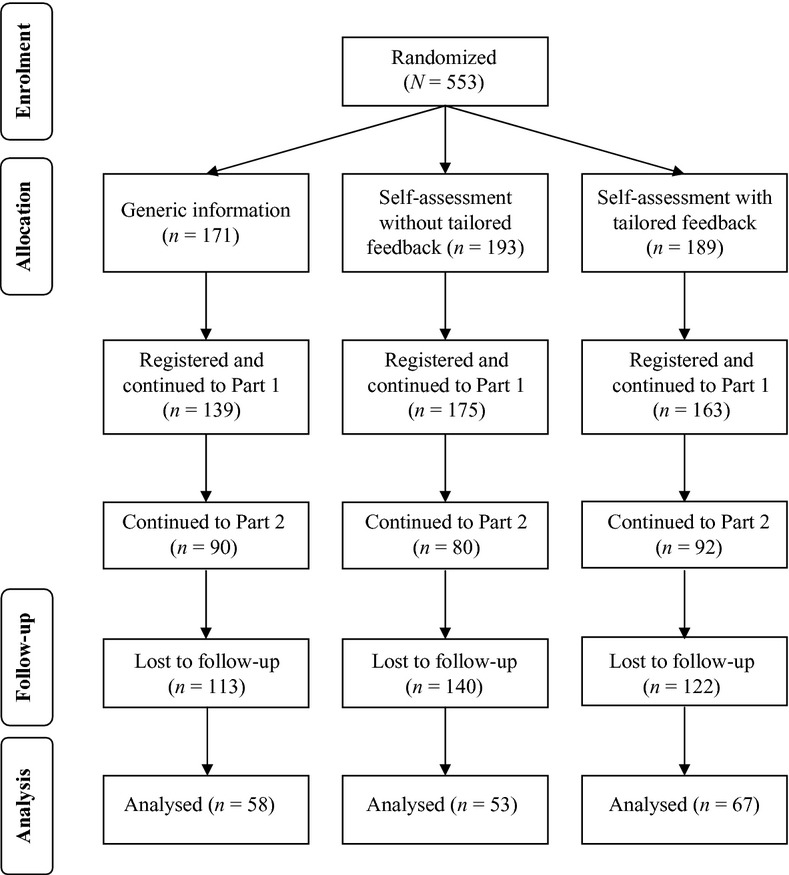
Flow of participants through each stage of the study.

In total, 553 participants registered for ‘Gut Instincts’. Of these, 178 (32%) saw both parts of the intervention and completed the follow-up measures (henceforth referred to as completers). Figure1 summarizes the flow of participants through each stage of the study. Sample characteristics of completers are summarized in Table 2006. There was a significant difference in dropout between the two self-assessment conditions, *z* = −1.68, *p* = .04. A higher proportion of participants tended to drop out of the self-assessment without tailored feedback condition (*n* = 140, 73%) than the tailored feedback condition (*n* = 122, 65%). The proportion of participants dropping out of the intervention did not differ significantly between the generic information (*n* = 113, 66%) and self-assessment without tailored feedback conditions, *z* = 1.34, *p* = .09, or between the generic information and tailored feedback conditions, *z* = −0.31, *p* = .38. Condition assignment was also significantly associated with return to Part 2 after dropping out in Part 1, *χ*^2^(2, *n* = 328) = 8.77, *p* = .01, *V* = 0.12. A higher proportion of participants returned to Part 2 in the generic information condition (*n* = 22, 13%) as compared to self-assessment without tailored feedback (*n* = 10, 5%), *χ*^2^(2, *n* = 222) = 8.49, *p* < .01.

**Table 3:** Sample characteristics, *n* (%), by condition

Characteristic	All (*n* = 178)	Generic information (*n* = 58)	Self-assessment without tailored feedback (*n* = 53)	Self-assessment with tailored feedback (*n* = 67)
Age, *M* (*SD*)	30.17 (11.66)	32.07 (11.89)	30.18 (12.10)	28.50 (10.99)
Sex: Female	139 (78)	53 (91)	37 (70)	49 (73)
Degree: Yes	158 (89)	50 (86)	47 (89)	61 (91)
Internet use, *M* (*SD*)	4.46 (3.41)	4.34 (2.71)	3.94 (2.42)	4.96 (4.45)

Note

Data are reported as *n* (%) unless specified otherwise. Percentages represent the proportion of the total number of participants that fell into each category.

On average, completers used ‘Gut Instincts’ for just over 20 min (excluding time spent completing study procedures and measures): 12 min on Part 1 and 10 min on Part 2. Total time spent using the intervention did not differ significantly between groups, *F*(2, 175) = 1.90, *p* = .15, partial η^2^ = .02. The majority of completers used both parts of the intervention in one session (*n* = 147, 83%). Completers who used each part of the intervention in two separate sessions (*n* = 31, 17%) left between 0 and 29 days between each part (*M* = 4.5 days).

The majority of participants in the tailored feedback condition gave the same answers to the self-assessment quizzes. Most participants reported experiencing both diarrhoea and constipation (*n* = 45, 67%) and saw further information about diet (*n* = 59, 88%), sleep (*n* = 62, 93%), stress (*n* = 63, 94%), diarrhoea (*n* = 50, 75%), and constipation (*n* = 42, 63%), but only a minority saw further information about physical activity (*n* = 16, 24%) and dealing with thoughts (*n* = 22, 33%). This meant that most participants in the tailored feedback condition saw similar information and advice to those in the other two non-tailored conditions.

### Satisfaction and engagement

Completers of all three conditions appeared to have positive perceptions of ‘Gut Instincts’, as operationalized by the PIPS scale (see Table 2011). There was a significant between-group difference in positive perceptions, *F*(2, 182) = 3.02, *p* = .05, partial η^2^ = .03. Positive perceptions were significantly higher in the generic information condition as compared to self-assessment without tailored feedback, *p* = .04, 95% CI [0.03, 1.70]. There were no significant differences between the generic information and tailored feedback conditions, *p* = .45, 95% CI [−0.38, 1.18], or the two self-assessment conditions, *p* = .36, 95% CI [−0.34, 1.28].

**Table 4:** Website satisfaction and evaluation scores at follow-up, *M* (*SD*)

Scale	Scale range	Generic information (*n* = 58)	Self-assessment without tailored feedback (*n* = 53)	Self-assessment with tailored Feedback (*n* = 67)
PIPS	0–10	7.07 (1.76)[Table-fn tf4-2]	6.20 (1.99)	6.67 (1.92)
Perceptions of personal relevance	1–5	3.78 (0.82)	3.42 (0.95)	3.53 (0.92)
Perceptions of self-assessment and goal setting	1–5	3.59 (0.83)	3.67 (0.87)	3.80 (0.80)
Engagement	1–5	3.74 (0.83)[Table-fn tf4-2]	3.36 (0.99)	3.68 (0.84)

*Note*. PIPS = Positive Intervention Perceptions Scale.

Indicates a significant difference between the generic information and self-assessment without tailored feedback conditions (*p* < .05, partial η^2^ = .03). ^b^Indicates a marginally significant difference between the generic information and self-assessment without tailored feedback conditions (*p* = .06, partial η^2^ = .03).

Scores on the three subscales of the Website Evaluation Questionnaire indicate that participants found ‘Gut Instincts’ personally relevant and engaging. There was a significant between-group difference for scores on the three subscales of the Website Evaluation Questionnaire, *F*(6, 360) = 6.21, *p* < .001, partial η^2^ = .09. There was a significant difference in engagement between the three conditions, *F*(2, 182) = 3.01, *p =* .05, partial η^2^ = .03. The generic information condition was rated as marginally more engaging and enjoyable to use as compared to self-assessment without tailored feedback, *p* = .06, 95% CI [−0.01, 0.77]. There were no significant differences between self-assessment with and without tailored feedback, *p* = .12, 95% CI [−0.06, 0.70], or the generic information and tailored feedback conditions, *p* = .92, 95% CI [−0.30, 0.43]. Group differences in perceptions of personal relevance were not quite significant, *F*(2, 182) = 2.41, *p* = .09, partial η^2^ = .03. Table 2011 indicates that the generic information condition was rated most personally relevant, followed by self-assessment with tailored feedback and self-assessment without tailored feedback, respectively. Perceptions of self-assessment and goal setting did not differ significantly between conditions, *F*(2, 182) = 1.12, *p* = .33, partial η^2^ = .01.

## Discussion

This study used mixed methods to investigate the effect of adding self-assessment and tailored feedback to generic information on participants’ engagement with an Internet-based health behaviour intervention. Our findings indicate that condition assignment had a small effect on participants’ engagement. Encouraging self-assessment without providing feedback was associated with higher dropout rates than self-assessment followed by tailored feedback. Self-assessment without providing feedback was also rated as less engaging and associated with fewer positive perceptions than generic information. Qualitative findings suggested that participants felt frustrated by answering self-assessment quizzes when they were not rewarded with individualized feedback based on their unique combination of responses. These findings suggest that the acceptability of self-assessment can be optimized by providing tailored feedback.

A recent Delphi study exploring optimal features of health-related websites identified both tailored feedback and self-assessment tools as important for optimizing the development of health-related websites (Schneider, van Osch, & de Vries, 2012). Our research adds to this guidance by showing the effect of providing these two features in combination. Our finding that self-assessment is more engaging when accompanied by tailored feedback is consistent with earlier qualitative research suggesting that self-monitoring features may be appreciated when accompanied by tailored feedback (Kerr, Murray, Stevenson, Gore, & Nazareth, 2006). This is also consistent with findings from a systematic review that suggested that self-monitoring components are most successful when they are accompanied by feedback on performance (Michie *et al*., 2009).

An unexpected finding was that no differences in engagement were observed between the tailored feedback and generic information conditions. This is counterintuitive given evidence showing that tailoring can be more engaging than providing generic information (e.g., Kreuter *et al*., 2000; Strecher *et al*., 2008). Several factors could explain why no differences were found between the tailored and generic information conditions in this study. First, most participants in our sample reported similar symptoms, behaviours, and thoughts in the self-assessment quizzes. This minimized the differences between the two conditions in terms of the information and advice provided. Thus, it may be that tailoring is more engaging when there are meaningful differences within the sample that necessitates the provision of tailored advice.

Second, the provision of tailored feedback may not have been obvious to participants. If the participants did not read the intervention content line by line, they may not have appreciated that the information and advice were tailored and therefore assumed that they were receiving the same information and advice as everybody else. The need to make personalization explicit to participants has been highlighted by other empirical studies examining the effects of tailoring (Dijkstra, 2005). There is also evidence to suggest that not only do participants need to be made aware that information has been tailored or personalized, they also need to be convinced about the benefits of tailoring. For example, Webb, Hendricks, and Brandon (2007) demonstrated that priming participants to perceive tailored or generic information as superior improved subsequent evaluations of personalized and generic self-help booklets for smoking cessation, respectively.

Third, the nature and quality of the tailored information itself may have adversely affected participants’ engagement with it. Findings from the qualitative pilot study indicated that tailored information was not always perceived positively by participants. Participants who disagreed with the tailored feedback may have perceived it as prescriptive or ‘nagging’ rather than helpful guidance. It is also possible that the self-assessment quizzes used to inform the tailored feedback did not reliably measure participants’ symptoms, thoughts, or behaviours. Indeed, participants disliked tailored feedback when it did not correspond with what they expected based on their answers to the self-assessment quiz or their own prior beliefs and experience. Tailored feedback was also disliked when participants felt they had not provided adequate information to inform it. These findings fit with the Elaboration Likelihood Model (Petty & Cacioppo, 1986), which argues that tailored information can result in disengagement if it is perceived to be unconvincing or contradictory to one’s prior beliefs (Petty, Rucker, Bizer, & Cacioppo, 2004). Tailored feedback is likely to be most engaging when it is based on adequate information about the participant and acknowledges their pre-existing beliefs.

Use of a controlled experimental manipulation enabled us to compare engagement with self-assessment without tailored feedback against engagement with self-assessment combined with tailored feedback, and engagement with generic information. This contrasts with meta-analyses of primary research which usually do not isolate the effect of individual design features or interventional studies where there are a number of differences between the intervention groups. The triangulation of self-report data, objective measures of usage, and qualitative data on subjective experiences meant that stronger inferences could be made about the level of engagement with each version of the intervention and the explanations underlying observed differences in engagement.

Limitations of this study include substantial attrition, which is consistent with other studies of Internet-based health interventions with minimal human support (Eysenbach, 2005; Khadjesari *et al*., 2011). This means that the study was underpowered to reliably detect group differences so we cannot rule out modest sized effects. We also cannot be confident that there is a real or meaningful difference between engagement with the self-assessment without tailored feedback and generic conditions given that the confidence interval for this finding contains zero. It is also possible that participants’ perceptions of the intervention may have been differentially influenced by whether or not they had taken time out between each part of the intervention to implement the practical advice. The findings of this study should therefore be considered exploratory rather than definitive. Reasons for dropout were not recorded. However, a design oversight may also have contributed to attrition in this study. It may not have been clear to participants that they needed to complete each part of the intervention in one sitting. If participants did not complete Part 1 before accessing Part 2, they missed out on any unfinished sections. Participants may have been discouraged from continuing with Part 2 once they realized that they could not revisit the first part of the intervention. The recruitment method may also have contributed to attrition in this study. The target population for this study were students with non-clinical or mild bowel complaints who may have been less motivated to complete both parts of the intervention.

Findings from the qualitative pilot study were used to infer differences between participants’ perceptions of each version of the intervention, but the context of the qualitative study may have influenced the thoughts and responses reported. For example, participants were specifically encouraged to express critical thoughts about the intervention. Participants were also interviewed by a young interviewer who portrayed herself as someone with no expert knowledge of the intervention or subject area. The specific request for critical comments from a non-expert interviewer may have artificially induced unfavourable perceptions of the self-assessment quizzes and tailored feedback.

The findings from this study have two practical implications for the optimal development of Internet-based behaviour change interventions. First, the use of phased or factorial experimental designs are vital for identifying how the implementation of different interactive design features, alone and in combination, is related to user engagement. Second, qualitative interviews during the development and evaluation of an intervention are essential for identifying the needs, preferences, and characteristics of the target population. These insights can be used to inform both future tailoring of intervention content and interpretation of subsequent quantitative evaluation.

This study moves a step closer towards the development of an evidence-informed model to guide the optimal design and implementation of online behavioural interventions by demonstrating the effect on engagement of design features alone (self-assessment without tailored feedback) and in combination (self-assessment with tailored feedback). Future research should seek to build on this foundation by examining the individual and combined effects of other interactive design features as well as identifying how the relationship between intervention design and user engagement is associated with change in health-related outcomes.

## Appendix: Website evaluation questionnaire

Items:

1. The information and advice provided by the website was of personal relevance to me
2. The website helped me to plan
3. The website kept my attention
4. The website helped me to think about my own behaviour
5. The website addressed my specific problems
6. The website was engaging
7. The information and advice provided by the website was appropriate for me
8. The website helped me to set goals to deal with my stomach or bowel problems
9. I found the website enjoyable to use

Notes:

Response options for all items range from 1 (*strongly disagree*) to 5 (*strongly agree*)
Perceptions of personal relevance subscale: Items 1, 5, 7
Perceptions of self-assessment and goal setting subscale: Items 2, 4, 8
Perceptions of engagement subscale: Items 3, 6, 9

## References

[b1] Bennett GG, Glasgow RE (2009). The delivery of public health interventions via the Internet: Actualising their potential. Annual Review of Public Health.

[b2] Braun V, Clarke V (2006). Using thematic analysis in psychology. Qualitative Research in Psychology.

[b3] Charmaz K (2006). Constructing grounded theory. A practical guide through qualitative analysis.

[b4] Collins LM, Baker TB, Mermelstein RJ, Piper ME, Jorenby DE, Smith SS, Fiore MC (2011). The multiphase optimisation strategy for engineering effective tobacco use interventions. Annals of Behavioral Medicine.

[b5] Collins LM, Murphy SA, Nair VN, Strecher VJ (2005). A strategy for optimising and evaluating behavioural interventions. Annals of Behavioral Medicine.

[b6] Danahar BG, Seeley JR (2009). Methodological issues in research on web-based behavioral interventions. Annals of Behavioral Medicine.

[b7] Danaher BG, McKay GH, Seeley JR (2005). The information architecture of behavior change websites. Journal of Medical Internet Research.

[b8] Dijkstra A (2005). Working mechanisms of computer-tailored health education: Evidence from smoking cessation. Health Education Research.

[b9] Dombrowski SU, Sniehotta FF, Avenell A, Johnston M, MacLennan G, Araújo-Soares V (2012). Identifying active ingredients in complex behavioral interventions for obese adults with obesity-related co-morbidities or additional risk factors for co-morbidities: A systematic review. Health Psychology Review.

[b10] Everitt HA, Moss-Morris RE, Sibelli A, Tapp L, Coleman NS, Yardley L, Little PS (2010). Management of irritable bowel syndrome in primary care: Feasibility randomised controlled trial of mebeverine, methylcellulose, placebo and a patient self-management cognitive behavioural therapy website (MIBS trial). BMC Gastroenterology.

[b11] Eysenbach G (2005). The law of attrition. Journal of Medical Internet Research.

[b12] Glasgow RE, Davidson KW, Dobkin PL, Ockene J, Spring B (2006). Practical behavioral trials to advance evidence-based behavioral medicine. Annals of Behavioral Medicine.

[b13] Kerr C, Murray E, Stevenson F, Gore C, Nazareth I (2006). Internet interventions for long-term conditions: Patient and caregiver quality criteria. Journal of Medical Internet Research.

[b14] Khadjesari Z, Murray E, Kalaitzaki E, White IR, McCambridge J, Thompson SG, Godfrey C (2011). Impact and costs of incentives to reduce attrition in online trials: Two randomized controlled trials. Journal of Medical Internet Research.

[b15] Kraft P, Yardley L (2009). Current issues and directions in Psychology and Health: What is the future of digital interventions for health behaviour change?. Psychology and Health.

[b16] Kreuter MW, Oswald D, Bull FC, Clark EM (2000). Are tailored health education materials always more effective than non-tailored materials?. Health Education Research.

[b17] Michie S, Abraham C, Whittington C, McAteer J, Gupta S (2009). Effective techniques in healthy eating and physical activity interventions: A meta-regression. Health Psychology.

[b18] Morrison LG (2012). How do users perceive and engage with Internet-based interventions to support health-related behaviour change? Doctoral dissertation.

[b19] Morrison LG, Yardley L, Powell J, Michie S (2012). What design features are used in effective e-Health interventions? A review using techniques from Critical Interpretive Synthesis. Telemedicine and e-Health.

[b20] Moss-Morris R, McAlpine L, Didsbury LP, Spence MJ (2010). A randomized controlled trial of a cognitive behavioural therapy-based self-management intervention for irritable bowel syndrome in primary care. Psychological Medicine.

[b21] Murray E, Burns J, See Tai S, Lai R, Nazareth I (2005). Interactive health communication applications for people with chronic disease. Cochrane Database of Systematic Reviews.

[b22] Nair V, Strecher V, Fagerlin A, Ubel P, Resnicow K, Murphy S, Zhang A (2008). Screening experiments and the use of fractional factorial designs in behavioural intervention research. American Journal of Public Health.

[b23] O’Brien HL, Toms EG (2008). What is user engagement? A conceptual framework for defining user engagement with technology. Journal of the American Society for Information Science and Technology.

[b24] Petty RE, Cacioppo JT (1986). The elaboration likelihood model of persuasion. Advances in Experimental Social Psychology.

[b25] Petty RE, Rucker D, Bizer G, Cacioppo JT, Seiter JS, Gass GH (2004). The elaboration likelihood model of persuasion. Perspectives on persuasion, social influence and compliance gaining.

[b26] Portnoy DB, Scott-Sheldon LAJ, Johnson BT, Carey MP (2008). Computer-delivered interventions for health promotion and behavioral risk reduction: A meta-analysis of 75 randomized controlled trials, 1988–2007. Preventive Medicine.

[b27] Schneider F, van Osch L, de Vries H (2012). Identifying factors for optimal development of health-related websites: A Delphi study among experts and potential future users. Journal of Medical Internet Research.

[b100] Spittaels H, De Bourdeaudhuij I, Brug J, Vandelanotte C (2007). Effectiveness of an online computer-tailored physical activity intervention in a real-life setting. Health Education Research.

[b28] Strauss AL, Corbin JM (1990). Basics of qualitative research: Grounded theory procedures and techniques.

[b29] Strecher VJ, McClure J, Alexander G, Chakraborty B, Nair V, Konkel J, Pormerleau O (2008). The role of engagement in a tailored web-based smoking cessation program: Randomized controlled trial. Journal of Medical Internet Research.

[b30] Wantland DJ, Portillo CJ, Holzemer WL, Slaughter R, McGhee EM (2004). The effectiveness of web-based vs. non-web-bases interventions: A meta-analysis of behavioral change outcomes. Journal of Medical Internet Research.

[b31] Webb MS, Hendricks PS, Brandon TH (2007). Expectancy priming of smoking cessation messages enhances the placebo effect of tailored interventions. Health Psychology.

[b32] Webb TL, Joseph J, Yardley L, Michie S (2010). Using the Internet to promote health behaviour change: A systematic review and meta-analysis of the impact of theoretical basis, use of behaviour change techniques, and mode of delivery on efficacy. Journal of Medical Internet Research.

[b33] Wright JH (2006). Cognitive behaviour therapy: Basic principles and recent advances. Focus.

[b34] Yang Y, Osmond A, Chen X, Weal M, Wills G, De Roure D, Yardley L (2009).

[b35] Yardley L, Smith JA (2008). Demonstrating validity in qualitative research. Qualitative psychology. A practical guide to research methods.

[b36] Yardley L, Bishop F, Willig C, Stainton-Rogers W (2007). Mixing qualitative and quantitative methods: A pragmatic approach. Qualitative research in psychology.

[b37] Yardley L, Joseph J, Michie S, Weal M, Wills G, Little P (2010). Evaluation of a web-based intervention providing tailored advice for self-management of minor respiratory symptoms: Exploratory randomized controlled trial. Journal of Medical Internet Research.

